# Bond Valence Sum Parameters for Analyzing Pyranopterin Tungsten Enzyme Structures

**DOI:** 10.3390/molecules30040871

**Published:** 2025-02-14

**Authors:** Jesse Lepluart, Martin L. Kirk

**Affiliations:** 1 Department of Chemistry and Chemical Biology, The University of New Mexico, MSC03 2060, 1 University of New Mexico, Albuquerque, NM 87131, USA; lepluart@unm.edu; 2 The Center for High Technology Materials, The University of New Mexico, Albuquerque, NM 87106, USA

**Keywords:** bond valence sum, pyranopterin, tungsten enzymes, X-ray crystallography, molybdenum enzymes, EXAFS, dithiolene

## Abstract

The determination of tungsten oxidation states and W–ligand bond lengths for pyranopterin tungsten enzymes can be negatively impacted by Fourier series termination effects and photodamage/photoreduction in the X-ray beam. As a result, a new set of bond valence sum (BVS) parameters have been derived from bond length data on W(+4) and W(+6) model compounds that were obtained from X-ray crystallography. These new W enzyme-specific BVS parameters have been used in the analysis of pyranopterin tungsten enzyme structural data. The results of this analysis indicate that there are potential issues with the enzyme crystal structures, including the number of ligating atoms to the tungsten atom, the W–ligand bond lengths, and the W oxidation state. We conclude that a BVS analysis of crystallographic and EXAFS structural data will help address these issues, and EXAFS should be more routinely employed in the determination of pyranopterin tungsten enzyme active site structures due to the increased accuracy of this technique for the determination of W–ligand bond distances.

## 1. Introduction

**Figure 1:**
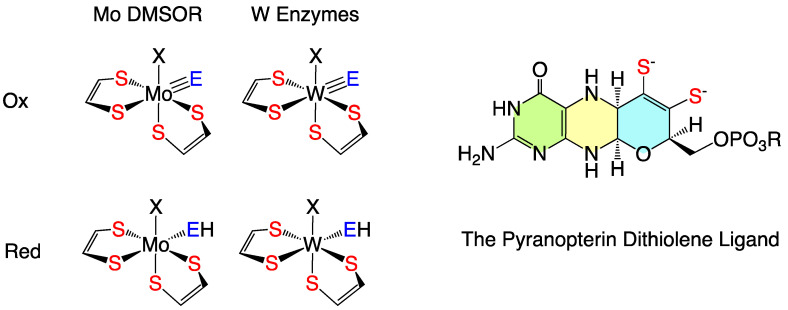
(**Left**) Generalized structures for oxidized (+6) and reduced (+4) active sites of the DMSO reductase family of pyranopterin Mo enzymes and for the pyranopterin W enzymes that are the focus of this manuscript. E = O, S; X = amino acid, OH_2_, OH^−^, no ligand. (**Right**) The pyranopterin dithiolene ligand present in these Mo and W enzymes.

The pyranopterin tungsten enzymes share many structural, functional, and catalytic similarities with members of the dimethyl sulfoxide reductase (DMSOR) family of pyranopterin molybdenum enzymes [1,2,3,4,5,6,7,8,9,10,11,12,13,14,15,16,17,18,19,20] (Figure 1). At structural parity, the chemical and reactivity profiles of molybdenum and tungsten compounds are very similar, with moderate differences in redox potentials and slight differences in bond strengths [13,21,22,23,24,25]. However, there are considerable differences in the nature of the organisms that express pyranopterin Mo and W enzymes [15,16]. Pyranopterin tungsten enzymes are dominantly expressed by extremophilic archaea and bacteria [16,26,27,28,29,30,31], though more recently tungsten enzyme-expressing bacteria have been discovered within the human gut microbiome [1]. The pyranopterin W enzymes [2,4,6,7,8,10,32] have been grouped into two enzyme families: the formate dehydrogenase family and the aldehyde oxidoreductase family. Formate dehydrogenase family enzymes include formate and formylmethanofuran dehydrogenases and acetylene hydratase, while the aldehyde oxidoreductase enzyme family contains aldehyde oxidoreductases and benzoyl-coenzyme A reductases. For both the Mo DMSOR family and pyranopterin W enzymes, the metal center is coordinated by two pyranopterin dithiolene (PDT) ligands [18,32,33,34], and these enzymes generally catalyze two-electron transfer reactions that are often coupled to oxygen atom or hydride transfer reactivity. Acetylene hydratase [2,35,36,37] and benzoyl-coenzyme A reductases represent two notable exceptions to the general reactivity profile of pyranopterin W enzymes, catalyzing the non-redox hydration of acetylene to acetaldehyde and the reductive degradation of aromatic molecules by a ring dearomatization reaction that has been proposed to occur without substrate binding to the W ion.

Determining the structure of pyranopterin tungsten enzyme active sites represents the starting point for understanding key structure–function relationships and the enzyme mechanism. Spectroscopic and computational studies, evaluated in the context of this structural information, are essential to develop electronic and geometric structure contributions to reactivity and enzymatic catalysis. As such, access to high-quality structural information obtained from X-ray crystallographic data dramatically influences our ability to properly understand the nature of their catalytic cycles. Detailed structural information is even more critical for understanding the mechanism of activity for metalloenzymes that are spectroscopically challenged, such as those that contain d^10^ metals or possess highly chromophoric redox cofactors that obscure the spectroscopic features of the active site. The pyranopterin W enzymes are solidly in this latter category, since most of these enzymes possess strongly absorbing [4Fe4S] or [2Fe2S] clusters that severely limit the general use of optical techniques such as electronic absorption, magnetic circular dichroism (MCD), and resonance Raman spectroscopies. Determining the active site structure of pyranopterin tungsten enzymes is complicated by the fact that tungsten is a heavy atom scatterer, and therefore the W–ligand bond distances that are determined by X-ray crystallography may be affected by Fourier series termination effects when the data are of limited resolution. This typically results in bond distances determined from X-ray crystallography being shorter than the actual W–ligand bond distance [38]. Additionally, photoreduction resulting from the high X-ray flux used during crystallographic data collection may also complicate the elucidation of the active site structure, compromising the ability to determine accurate W–ligand bond lengths and the W oxidation state.

Extended X-ray absorption fine structure, or EXAFS, represents a complementary method for determining W–ligand bond lengths at the active sites of these enzymes. In fact, for metal–ligand bond distances involving heavy atom scatterers, EXAFS has proven to be an excellent method for obtaining first coordination sphere bond distances that are more accurate than those determined by X-ray crystallography [39]. Oxidation state information may be obtained from an analysis of the X-ray absorption near-edge spectroscopy (XANES) edge energy. Additionally, the bond valence sum (BVS) approach [40,41,42,43,44,45] can be used to determine metal–ligand bond distances when the oxidation state of the metal ion is known or, conversely, the oxidation state can be assessed using known metal–ligand bond distances. Thus, the BVS approach can be used in conjunction with protein X-ray crystallography and EXAFS/XANES results to provide a more comprehensive description of active site geometric structure and the metal ion oxidation state, particularly when the metal ion is coordinated by both light and heavy atom donors. This approach is particularly useful when structural data have been obtained on enzyme samples that may contain off-pathway or damaged structures. Here, we have developed a new set of BVS parameters that have been specifically optimized for analyzing the structure of pyranopterin tungsten enzyme active sites, evaluated the utility of these parameters using small molecule analogs of pyranopterin W enzymes, and used this information to evaluate available crystallographic and EXAFS results on pyranopterin tungsten enzymes to provide insight into the structures obtained using these techniques.

## 2. Results

### 2.1. New BVS Parameters for Pyranopterin Tungsten Enzymes

In the bond valence sum approach, the sum of the individual bond valences (*s_ij_*) of individual bonds between the metal ion (*i*) and its coordinated ligands (*j*) is equal to the formal valence $V_{i}$ (i.e., the oxidation state) of the metal ion according to [45]

(1) $$V_{i}=\sum_{j}s_{ij}$$

where the individual bond valences are defined as

(2) $$s_{ij}=exp\left(\left(R_{0}-R_{ij}\right)/B\right)$$

Here, the $R_{ij}$ are the experimentally observed metal–ligand bond lengths, and $R_{0}$ and B are empirically derived bond valence parameters. The *R*_0_ parameter is the unit valence bond length, while the B parameter, which is frequently fixed at a value of *B* = 0.37 [46], can reflect the degree of metal–ligand covalency. Thus, *B* describes the relationship between changes in charge and changes in bond length [45]. Bond lengths shorter than $R_{0}$ will contribute a greater individual bond valence, or a greater amount of electron density or negative charge to offset the increased nuclear–nuclear repulsion. Smaller values of B are reflective of more ionic metal–ligand bonding interactions, while B values greater than 0.37 describe more covalent interactions. It has been observed that B values can be significantly higher than 0.37 for bonds involving heavier metals, such as lanthanides and actinides [47]. It is believed that larger *B* values are also reflective of softer (i.e., more covalent) bonding interactions [48].

BVS parameters have traditionally been derived from structural data sets that consist of ten or more homoleptic compounds [46]. Since the *B* parameter is often fixed at a value of 0.37, the determination of the *R*_0_ parameter under this constraint is trivial, and *R*_0_ adopts a value close to the average bond length observed in the compounds from which structural data are obtained. However, using this approach to derive new BVS parameters for pyranopterin tungsten enzymes is complicated by the dearth of relevant, well-characterized homoleptic small molecule analog complexes. Thorp et al. [43] described how this can be circumvented by deriving parameters from heteroleptic compounds, with a special emphasis on ensuring as much similarity as possible between the models from which BVS parameters are derived and the enzymes for which these parameters are to be applied. Holm and coworkers have synthesized a large number of heteroleptic W^VI^ and W^IV^ model compounds with pyranopterin W enzyme-relevant bis-dithiolene ligation that fit these criteria [13,22,24,25,49,50,51,52,53,54], and these model compounds are listed in Table 1.

This library of W^VI^ and W^IV^ bis-dithiolene model compounds have been structurally characterized by X-ray crystallography (.cif filenames are provided in Table 1), and the listed compounds were originally designed as structural models for the oxidized and reduced active sites of pyranopterin W enzymes. In addition to the two bidentate dithiolene ligands that are chelated to the W ion in each of these complexes, a combination of oxo, thiolate, selenolate, phenolate, and carbonyl ligands are also found coordinated to the W center. High-resolution X-ray crystal structures have been published for eight W(+6) and sixteen W(+4) compounds, each with a unique chemical formula. However, for some of these compounds, there are multiple molecules within a single crystallographic unit cell, each with unique but similar tungsten–ligand bond lengths and angles. This results in nine unique W(+6) sites and twenty-two unique W(+4) sites. The tungsten–ligand bond lengths obtained from the crystallographic coordinates of the compounds in Table 1 yield a total of forty-six W(+6)-S bonds, seven W(+6)-O bonds, one W(+6)-Se bond, ninety-six W(+4)-S bonds, six W(+4)-O bonds, seven W(+4)-Se bonds, and fifteen W(+4)-CO bonds. This has allowed us to develop and refine BVS parameters specific to the W(+6) and W(+4) oxidation states using Equations (1) and (2) (see Section 4, Materials and Methods). These W–ligand BVS parameters are provided in Table 2, where they are compared with earlier, more generalized parameter sets that were not determined specifically for pyranopterin tungsten enzymes. Importantly, these enzyme-relevant BVS parameters can now be used to evaluate available pyranopterin tungsten enzyme X-ray crystallographic data and EXAFS-derived active site structures. Although the small molecule structural data set is somewhat limited, we believe this limitation is ameliorated by the high degree of structural relevance of the model compounds to the enzyme active sites.

### 2.2. Comparison of W Enzyme-Specific BVS Parameters with General W BVS Parameters

**Figure 2:**
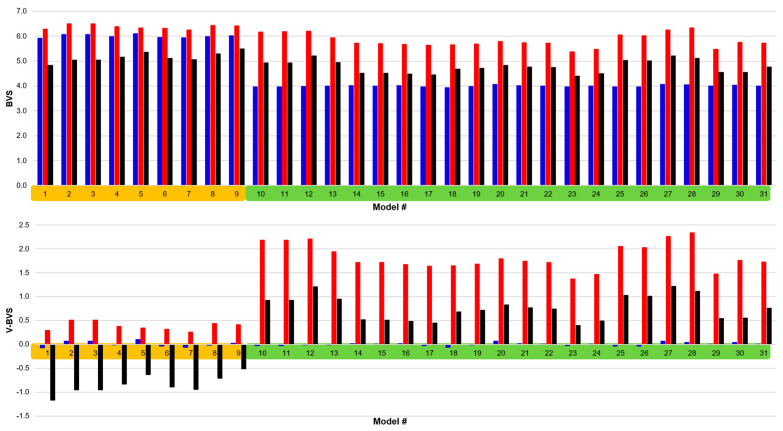
Application of Set A (red), Set B (black), and NEW (blue) BVS parameters to the library of W small molecule analog compounds (**top**), and the variance (**bottom**) between the known oxidation states of the models and the bond valence sum values derived from Equation (1). Complexes 1–9 (orange) are W(VI) models, while complexes 10–31 (green) are W(IV) models. Complexes 14–22 and 30–31 contain W-CO bonds, for which there are no previously published W-C BVS parameters.

We have applied the three sets of BVS parameters in Table 2 to the model compounds listed in Table 1, and the results are presented in Figure 2. Here, it is clearly observed that neither the Set A BVS parameters [55], which include tungsten parameters derived by two different groups [56,57], nor the Set B parameters that employ theoretically derived parameters [58] provide accurate oxidation states for the model compounds listed in Table 1. The Set A parameters include those that are oxidation state-specific for W-O bonds in addition to W-S and W-Se BVS parameters that are not tungsten oxidation state-specific. The Set B BVS parameters for W-S and W-O bonds are independent of the W oxidation state. Although the model compounds in Table 1 are known to be either in the W(+4) or W(+6) oxidation state, the use of Set A parameters always yields a BVS near +6, while the Set B BVS parameters yields results near +5 (Figure 2). A useful measure of the accuracy for a given BVS parameter set is the variance, which describes the difference between the computed *V_i_* and expected (integer) valence or oxidation state. A smaller variance using a given BVS parameter set is reflective of more accurate BVS parameters. Although the Set A parameters compute the true valence of the W(+6) enzyme model compounds with some degree of accuracy (maximum variance of +0.51), they are quite inaccurate when applied to the W(+4) models (minimum variance of +1.37, maximum variance of +2.34). The variance in *V_i_* using the Set B parameters range from −1.16 to +1.22, with a minimum variance absolute value of 0.40. Thus, neither the BVS parameters from Set A nor Set B can be used to accurately determine an unknown tungsten oxidation state for the model compounds in Table 1 or, by inference, the oxidation state or W–ligand bond lengths in pyranopterin tungsten enzymes. In marked contrast, the NEW BVS parameter set derived here accurately reflects the known oxidation state assignment for all the relevant W model complexes listed in Table 1. A comparison of the NEW BVS parameters with the general Set A and Set B parameters is given in Table 2. The NEW *R*_0_ parameters that we have derived are similar to the previously determined *R*_0_ values, since unit valence bond lengths are not expected to change significantly between similar compounds. The NEW *B* parameters differ from the standard value of 0.37 and vary as a function of oxidation state and W–ligand bond type. We note that the NEW *B* values are generally higher than the *B* parameters for Set A or Set B. This likely reflects the highly covalent nature of the W-L bonds in the model compounds listed in Table 1. Additionally, we note what we believe to be the first derived tungsten-carbon BVS parameter set, though it should more accurately be considered a W-CO parameter set due to the unique aspects of metal–carbonyl backbonding.

**Figure 3:**
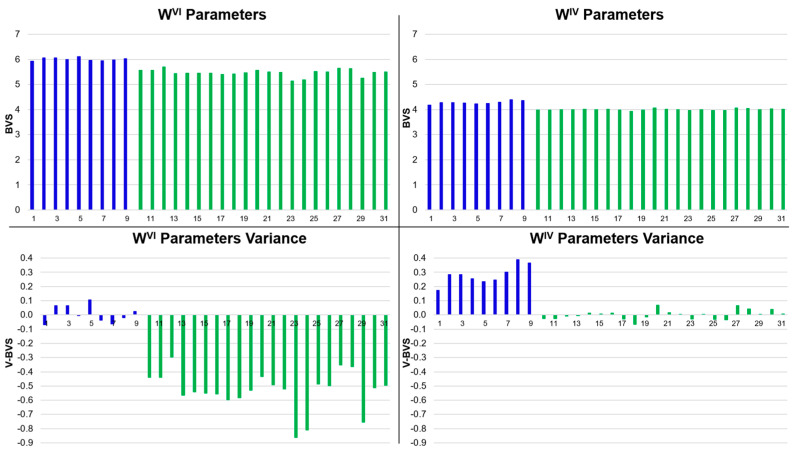
BVS results of applying NEW W(+6) BVS parameters (**upper left**) and NEW W(+4) BVS parameters (**upper right**) to all the compounds found in Table 1 regardless of the known tungsten oxidation state for these compounds. The BVS deviation is depicted in the two bottom charts in the figure. (**Bottom left**) Deviation in the BVS results from the expected value of +6. (**Bottom right**) Deviation in the BVS results from the expected value of +4. The known W(+6) compounds are represented by blue bars, while the known W(4) compounds are represented by green bars.

Applying all three parameter sets (A, B, and NEW) to the full library of model compounds in Table 1 demonstrates the accuracy of the NEW parameters in describing known differences in tungsten oxidation state. The accuracy of the NEW BVS parameter set is clearly shown in Figure 3. When the NEW W(+6) parameters are applied appropriately to known W(+6) compounds, or NEW W(+4) BVS parameters to known W(+4) compounds, the BVS variance of the NEW parameters is remarkably small (Figure 3). We suggest that a more nuanced approach is required if the NEW BVS parameters are to be used to determine an unknown tungsten oxidation state. When the NEW W(+6) parameters are indiscriminately applied to all the compounds in Table 1, the BVS results are always near +6 (Figure 3). Similarly, when the NEW W(+4) parameters are universally applied to all the compounds in Table 1, the BVS results are always near +4. This is due to the extremely covalent nature of W–S bonding which renders the effective nuclear charge at the W ion to be minimally affected as a function of the oxidation state. This means that the bond length changes as a function of oxidation state are quite subtle, even when there is a two-electron difference in the tungsten oxidation state. Although the NEW BVS parameters must be used with care, the variance of the computed BVS from the expected values of +4 and +6 clearly demonstrates that the NEW BVS parameters are more precise (smaller variance) when applied to compounds of the same (or correct) oxidation state. Thus, to determine an unknown oxidation state in the enzymes, both NEW parameter sets should be applied simultaneously, and the parameter set that produces the smallest variance reveals the likely oxidation state of tungsten ion in the enzyme active site.

## 3. Discussion

When compared to pyranopterin molybdenum enzyme bond length data, the published active site structural data for tungsten enzymes are relatively sparse. In this work, we have evaluated W–ligand bond length data and likely oxidation state assignments associated with 22 tungsten enzyme crystal structures [27,38,59,60,61,62,63,64,65]. These active site data represent seven different pyranopterin tungsten enzymes (acetylene hydratase (AH); dimethyl sulfoxide reductase (DMSOR); formate dehydrogenase (FDH); formylmethanofuran dehydrogenase (FMFDH); benzoyl-CoA reductase (BCoAR); aldehyde oxidoreductase (AOR); and formaldehyde ferredoxin oxidoreductase (FFOR)). The pyranopterin tungsten active site structural data that were subjected to our BVS analyses were obtained from the Protein Data Bank. In addition to the X-ray crystallographic data, we have included two sets of EXAFS-derived W enzyme bond length data in our BVS analysis [59,64].

**Figure 4:**
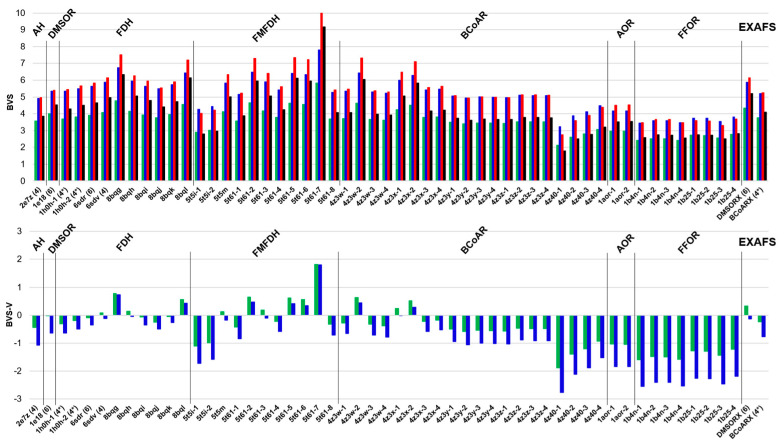
Application of the BVS approach to tungsten enzyme active site bond length data. (**Top**) BVS of NEW W(+6) (blue), NEW W(+4) (green), Set A (red), and Set B (black) parameters. (**Bottom**) Variance of NEW W(+6) (blue) and NEW W(+4) (green) *Vi* parameters. The W enzymes are labeled using their PDB identifications (first four characters); hyphen-number notation indexes indicate multiple active sites; the number in parentheses indicates explicit tungsten oxidation state assignment by the original authors; and a starred number in parentheses (*) indicates an assumed tungsten oxidation state based on preparation methods. Two EXAFS-derived bond length data sets are also included (on the far right of the charts). Only W-O, W-S, and W-Se bonds are assumed in this analysis.

It was previously noted that calculated *V_i_* values within ±0.25 for Mn, Fe, Cu, and Zn 3d transition metal complexes were deemed reliable [44]. Clearly, this is not the case when the Set A and Set B parameters are applied to W model compounds and W sites in enzymes (Figure 4). Application of the BVS approach to enzyme active site structural data using the NEW BVS parameter set generated in this work reveals significantly greater variances compared to the variances determined for the biomimetic compounds in Table 1 (Figure 4). We observe that the BVS variance for many of the W active sites in published X-ray crystal structures are greater than one valence unit (|*V_i_* − *V*| > 1), and this indicates the possibility of either X-ray-induced photodamage/photoreduction or a mischaracterization of type and/or number of ligand atoms bound to the W ion. The majority of our calculated BVS *V_i_* values are lower than what would be expected for the expected oxidation state, and we believe this to be a reflection of either longer W–ligand bonds in the enzymes or photoreduction of the W ion. Few of the W sites in these enzymes have been assigned an explicit oxidation state by the original authors. Two crystal structures explicitly stated the W ion to be in the W(+6) oxidation state (1E18 DMSOR and 6SDR FDH), but our BVS analysis suggests that these are more likely to be W(+4) sites. Four active site structures determined by X-ray crystallography yield BVS results with apparent minimal variance: 6SDV FDH, 5T5M and 5T61-3 FMFDH, and 4Z3X-1 BCoAR. However, specific individual W–ligand bonds at these enzyme active sites are either significantly longer or shorter than the unit bond length. Thus, the small calculated variance is a reflection of individual bond valence (*s_ij_*) variances effectively cancelling each other out in the BVS (*V_i_*). Below, we highlight our observations regarding the application of a BVS approach with enzyme-specific BVS parameters to a few specific pyranopterin enzyme active site structures.

Presently, there are reliable combined crystal structure and EXAFS data for only two tungsten enzymes: benzoyl-CoA reductase (BCoAR) [37] and dimethyl sulfoxide reductase (DMSOR) [59]. The W-DMSOR enzyme, however, is not a naturally occurring tungsten enzyme but was obtained from protein grown in vitro on a tungstate-enriched substrate, effectively substituting tungsten for the naturally occurring molybdenum ion in the active site. The results of this BVS study applied to the W-DMSOR enzyme crystal structure bond length data suggest a reduced W(+4) active site. Here, there is a calculated −0.01 valence unit variance using our W(+4) parameters and a −0.64 valence unit variance using our W(+6) parameters (i.e., the NEW parameter set). However, the results of our BVS analysis applied to EXAFS data suggest an oxidized W(+6) active site (−0.13 valence unit variance with W(+6) parameters, +0.33 valence unit variance with W(+4) parameters). The original authors deliberately prepared and obtained data on this enzyme in the W(+6) oxidized state. In marked contrast to the W-DMSOR data, we used 20 unique active sites using available BCoAR crystal structure data, and most of these active sites are in complex with varying substrates. Only the 4Z40 structure represents an as-isolated enzyme. Our BVS analyses of these BCoAR data suggest the majority of these active sites are in the W(+4) oxidation states. Our BVS analysis of the as-isolated BCoAR EXAFS data similarly suggests a W(+4) oxidation state (−0.77 variance with W(+6) parameters, −0.23 variance with W(+4) parameters). This was obtained by treating the sixth (terminal) ligand as an oxygen donor, but if this sixth ligand is assigned as a carbonyl, as the original authors have suggested may be possible, the calculated variance with W(+4) parameters becomes +0.05. The improvement in BVS variance supports the assignment of a carbonyl ligand bound to the W(+4) site. As-isolated BCoAR is indicated by the original authors to be in the reduced W(+4) state.

Collectively, our BVS results using the NEW parameter set derived in this work suggest that EXAFS provides highly accurate bond length data for pyranopterin tungsten enzymes and should be used in conjunction with X-ray crystallography and BVS analyses to provide a complete description of these enzyme active sites to yield W–ligand bond length and oxidation state information. When analyzing EXAFS data, there can be some ambiguity regarding the exact number or identity of the atoms that are coordinated to the W ion. The BVS approach can assist in resolving these issues. We show that W–ligand bond lengths derived from protein X-ray crystal structure data should be viewed with caution due to the potential for sample photodamage and metal ion photoreduction. Again, the BVS approach can be used to address the presence and even the degree of photodamage to the sample and may even assist in determining the nature of unknown ligand donor atoms.

Our bond valence sum analysis using these newly derived BVS parameters was applied to two different structural models for the acetylene hydratase active site: one with series termination effects on the observed W–O bond accounted for and corrected (W-O = 2.25 Å), and one without (W-O = 2.04 Å), with all other bonds lengths retained from the crystal structure. In both cases, our BVS results suggest a W(+4) active site for acetylene hydratase (−1.08 variance with W(+6) parameters, −0.43 variance with W(+4) parameters for the corrected model; −0.80 variance with W(+6) parameters, −0.22 variance with W(+4) parameters for the uncorrected model). Interestingly, there is less observed variance using the X-ray crystallographic bond length data that does not correct for series termination effects. However, our BVS analysis still supports the original crystallographic assignment of a W(+4) ion at the acetylene hydratase active site. Finally, we note that there are limitations that affect how these W enzyme-specific BVS parameters should be employed in an analysis of pyranopterin W enzyme structures. The first is that it is difficult to distinguish the true nature of X in M-X bonds in enzymes since the size of X is reflected in the value of *R*_0_ [43]. This limitation is essentially the same as that observed in EXAFS analyses, where it is difficult to determine the nature of X when the coordinated atoms have similar atomic masses [43]. The second issue is one that we already mention in our study. Namely, in structures that possess highly covalent M–X bonds, the M–X bond lengths do not tend to change dramatically as a function of oxidation state.

## 4. Materials and Methods

Most published BVS parameters are derived from bond length data of ten or more homoleptic compounds [46]. Studies have shown that varying the B parameter for most commonly observed element pairs does not result in significant differences in BVS results. The B parameter is thus traditionally fixed at a value of 0.37, making optimizing the $R_{0}$ parameter somewhat trivial. For this work, however, it was determined that B parameters would also be optimized to account for varying covalency. Deriving new parameters from heteroleptic compounds requires the simultaneous optimization of multiple variables to minimize variance. The function minimized for this work was the sum of chi-squared for each oxidation state of tungsten:

(3) $$\sum X^{2}=\sum \frac{{\left(BVS-V\right)}^{2}}{V}$$

where V is the formal tungsten oxidation state of either +4 or +6. Thus, optimizing parameters for W(+6) compounds involved minimizing a six-variable function ($R_{0}$ and B each for W^VI^-O, W^VI^-S, and W^VI^-Se bonds), while optimizing parameters for W(+4) compounds involved minimizing an eight-variable function ($R_{0}$ and B each for W^IV^-O, W^IV^-S, W^IV^-Se, W^IV^-C≡O bonds). Minimization of the sum of chi-squared equations was performed using the *fminsearch* MatLab (v. R2019a) Simplex routine. The *fminsearch* function is not guaranteed to locate the global minimum of a function and can converge to any number of local minima depending on the initial values of the variables. Also, there is no guarantee that the $R_{0}$ and B parameters at the global or local minima are chemically meaningful or relevant. $R_{0}$ and B are expected to fall within certain ranges: $R_{0}$ should be comparable to an average bond length, while B should fall between 0.25 and 0.65 [58]. Often, the minimization converged with one or more parameters falling well outside their expected range. Optimization of the new parameters was therefore a delicate balance of minimizing variance while still obtaining realistic values. This was achieved in a two-step optimization process where initially some of the variables were allowed to optimize while others remained fixed and then the remaining variables were optimized in a second step. The initial $R_{0}$ values were given as the average of the observed bond lengths, while the initial B parameters were set as 0.37. The new parameters (NEW) are included, along with those from Set A and Set B, in Table 2. For W(+6) parameters, all $R_{0}$ and W^VI^-O B were optimized during the first iteration and W^VI^-S B and W^VI^-Se B in the second. For the W(+4) parameters, all but W^IV^-C≡O B were optimized during the first iteration. Ultimately, our optimized BVS parameters were obtained from function minimizations with the fewest variables held constant in the first minimization step. The final optimized BVS parameters do not produce a true global minimum in the sum of chi-squared function, but rather, they represent a local minimum, in which the parameters adopt realistic or chemically intuitive values. The final sum of chi-squared for the W(4) parameters is 0.005604, and the final sum of chi-squared for the W(6) parameters is 0.003360.

The model compounds that we employed here to construct *enzyme-specific* BVS parameters were selected based on their high structural relevance to pyranopterin tungsten enzyme active sites. The primary determining criterion was the inclusion of two bidentate dithiolene ligands since a bis-PDT coordination environment is present in all known tungsten enzyme active sites. Second, the model compounds we chose possessed additional ligands that specifically mimic other ligands bound to the W ion in the enzymes. Namely, these ligands include alkoxides, thiolates, selenolates, oxidos, sulfidos, and carbonyls representing the serine, aspartate, cysteine, and selenocysteine amino acids that bind to W in the enzymes, in addition to coordinated CO, terminal oxo, and terminal sulfido ligands. Complexes not meeting these criteria, such as those with nitrogen or phosphorus atoms coordinated directly to W, or employing W ions in the +2 or +5 oxidation state (which tend to dimerize), were not included due to their irrelevance to enzyme active site oxidation states and/or geometries. In summary, our new BVS parameter sets were derived for the specific and explicit purpose of analyzing pyranopterin tungsten enzyme active sites, hence the increased selectivity in choosing well-characterized model compounds from which to derive these new BVS parameters.

## 5. Conclusions

Unfortunately, EXAFS data for pyranopterin tungsten enzymes are quite sparse [9,30,66]. Early EXAFS data on pyranopterin tungsten enzymes [9,30,66] were analyzed using fewer sulfur donors coordinated to the metal ion. These early EXAFS data were collected prior to the discovery of a second PDT ligand in the X-ray structure of the tungsten enzyme aldehyde ferredoxin oxidoreductase [27]. However, when applying BVS analyses to EXAFS-derived structures with two coordinated pyranopterin dithiolene ligands, the EXAFS results are in good agreement with our BVS analyses. Given the uncertainty in crystallographically derived W–ligand bond lengths and oxidation state assignments, we suggest that crystallographic data on pyranopterin tungsten enzymes be accompanied by EXAFS data and a BVS analysis. Bond distances in covalent systems are less sensitive to changes in oxidation state than they are in ionic systems. Since pyranopterin tungsten enzyme active sites possess many highly covalent W–ligand bonds, this requires a careful application of the BVS parameters provided here when making W oxidation state assignments. We acknowledge that there are limitations due to the non-atomic resolution of the pyranopterin tungsten enzyme X-ray structures, but photodamage and photoreduction can occur in X-ray crystallographic studies of metalloenzyme active sites [67]. The large variances that we observe in our BVS analysis indicate that the structures determined by protein X-ray crystallography may not fully reflect the nature of the catalytic active site in the enzymes. We note that X-ray structures with fully intact active sites typically exhibit longer than expected W–ligand bond lengths. Considering the generally poor bond length resolution of protein crystallography compared with EXAFS data, we believe that these distances should be used with caution when making oxidation state assignments based solely on crystallographic data.

## Figures and Tables

**Table 1:** Compounds used for determining bond valence sum parameters.

Compound Number	.cif Filename	Chemical Formula
1	ic062441a-1	W(S_2_C_2_Me_2_)_2_(SPh(Pr^i^)_3_)_2_
2	ic9903329-9-1	W(S_2_C_2_Ph_2_)_3_
3	ic9903329-9-2	W(S_2_C_2_Ph_2_)_3_
4	ic062441a-4	[NEt_4_][W(O)(S_2_C_2_Me_2_)_2_(SBu^t^)]
5	ic062441a-7	[NEt_4_][W(O)(S_2_C_2_Me_2_)_2_(SeBu^t^)]
6	ic062441a-12	[NEt_4_][W(O)(S_2_C_2_Me_2_)_2_(S-1-Ad)]
7	ic062441a-13	[NEt_4_][W(OSiPh_3_)(S_2_C_2_Me_2_)_2_(S-1-Ad)]
8	ic010421x-8	[NEt_4_][W(S)(OPh)(S_2_C_2_Me_2_)_2_]
9	ic010421x-9	[NEt_4_]_2_[W(O)_2_(S_2_C_2_Me_2_)_2_]
10	ic062441a-2	[NEt_4_][W(S-Bu^t^)(S_2_C_2_Me_2_)_2_]
11	ic062441a-9	[NEt_4_][W(S_2_C_2_Me_2_)_2_(S-1-Ad)]
12	ic062441a-5	[NEt_4_][W(S_2_C_2_Me_2_)_2_(SeBu^t^)]
13	ic991153u-1	[NEt_4_][W(OPh)(S_2_C_2_Me_2_)_2_]
14	ic991153u-2-1	[NEt_4_][W(CO)(SPh)(S_2_C_2_Me_2_)_2_]
15	ic991153u-2-2	[NEt_4_][W(CO)(SPh)(S_2_C_2_Me_2_)_2_]
16	ic991153u-2-3	[NEt_4_][W(CO)(SPh)(S_2_C_2_Me_2_)_2_]
17	ic991153u-3	[NEt_4_][W(CO)(SPh(Pr^i^)_3_)(S_2_C_2_Me_2_)_2_]
18	ic991153u-4-1	[NEt_4_][W(CO)(SePh)(S_2_C_2_Me_2_)_2_]
19	ic991153u-4-2	[NEt_4_][W(CO)(SePh)(S_2_C_2_Me_2_)_2_]
20	ic991153u-4-3	[NEt_4_][W(CO)(SePh)(S_2_C_2_Me_2_)_2_]
21	ic991153u-4-4	[NEt_4_][W(CO)(SePh)(S_2_C_2_Me_2_)_2_]
22	ic991153u-5	[NEt_4_][W(CO)(SePh(Pr^i^)_3_)(S_2_C_2_Me_2_)_2_]
23	ic9903329-1	W(CO)_2_(S_2_C_2_Me_2_)_2_
24	ic9903329-2	W(CO)_2_(S_2_C_2_Ph_2_)_2_
25	ic9903329-3-1	[NEt_4_]_2_[W(O)(S_2_C_2_Ph_2_)_2_]
26	ic9903329-3-2	[NEt_4_]_2_[W(O)(S_2_C_2_Ph_2_)_2_]
27	ic9903329-5	[NEt_4_][W(O)(MeS_2_C_2_Ph_2_)(S_2_C_2_Ph_2_)
28	ic9903329-6	[NEt_4_]_2_[W(S)(S_2_C_2_Ph_2_)_2_]
29	ic010421x-1	[NEt_4_][W(O_2_CPh)(S_2_C_2_Me_2_)_2_]
30	ic010421x-2	[NEt_4_][W(CO)(S-2-Ad)(S_2_C_2_Me_2_)_2_]
31	ic010421x-3	[NEt_4_][W(CO)(Se-2-Ad)(S_2_C_2_Me_2_)_2_]

**Table 2:** Tungsten bond valence sum parameters.

W–Ligand Bond	Set A (*R*_0_, *B*)	Set B (*R*_0_, *B*)	NEW Set (*R*_0_, *B*)
W^IV^-O W^VI^-O	1.851, 0.370	1.901, 0.303	1.804, 0.489
	1.896 ^a^, 0.280 ^a^	1.901 ^a^, 0.303 ^a^	1.889, 0.375
W^IV^-S W^VI^-S	2.390 ^a^, 0.370 ^a^	2.307 ^a^, 0.3027 ^a^	2.207, 0.456
	2.390 ^a^, 0.370 ^a^	2.307 ^a^, 0.3027 ^a^	2.366, 0.515
W^IV^-Se W^VI^-Se	2.510, 0.370	N/A	2.336, 0.418
	2.510, 0.370	N/A	2.514 ^b^, 0.401 ^b^
W^IV^-CO W^VI^-CO	N/A	N/A	1.843, 0.371
	N/A	N/A	N/A

^a^ Parameters that do not depend on oxidation state. ^b^ Parameters obtained from a single bond length. Note that most NEW Set parameters were determined from an analysis of at least six unique bonds. Bond types without previously published parameters or with insufficient data from which to derive new parameters are indicated by N/A (not-applicable).

## Data Availability

The data used in this manuscript may be found in the cited literature.
